# Defending public interests in private lands: compliance, costs and potential environmental consequences of the Brazilian Forest Code in Mato Grosso

**DOI:** 10.1098/rstb.2012.0160

**Published:** 2013-06-05

**Authors:** Claudia M. Stickler, Daniel C. Nepstad, Andrea A. Azevedo, David G. McGrath

**Affiliations:** 1 International Program, Amazon Environmental Research Institute, San Francisco, CA 94110, USA; 2 Instituto de Pesquisa Ambiental da Amazônia, Brasilia, Distrito Federal 71503-505, Brazil; 3 Woods Hole Research Center, Falmouth, MA 02540, USA

**Keywords:** deforestation, forest governance, land-use regulation, ecosystem services, environmental policy, Amazon

## Abstract

Land-use regulations are a critical component of forest governance and conservation strategies, but their effectiveness in shaping landholder behaviour is poorly understood. We conducted a spatial and temporal analysis of the Brazilian Forest Code (BFC) to understand the patterns of regulatory compliance over time and across changes in the policy, and the implications of these compliance patterns for the perceived costs to landholders and environmental performance of agricultural landscapes in the southern Amazon state of Mato Grosso. Landholdings tended to remain in compliance or not according to their status at the beginning of the study period. The perceived economic burden of BFC compliance on soya bean and beef producers (US$3–5.6 billion in net present value of the land) may in part explain the massive, successful campaign launched by the farm lobby to change the BFC. The ecological benefits of compliance (e.g. greater connectivity and carbon) with the BFC are diffuse and do not compete effectively with the economic benefits of non-compliance that are perceived by landholders. Volatile regulation of land-use decisions that affect billions in economic rent that could be captured is an inadequate forest governance instrument; effectiveness of such regulations may increase when implemented in tandem with positive incentives for forest conservation.

## 1. Introduction

Tropical forests regulate energy and water flow, store 260 GtC (billion tons of carbon) in their trees [1], are rich in biodiversity and are home to more than a 1000 indigenous cultures [2]. They are also rapidly ceding space through conversion to crops, livestock and agrarian reform settlements. Most public policies for defending public interests in tropical forests have focused on protected areas that prohibit most types of economic activities and that have met with partial success [3,4]. Policies are also needed for conserving forests in agricultural and livestock landscapes, and on lands that are privately owned. Some policies have been developed for creating positive incentives for forest maintenance or restoration through payments for ecosystem services [5]. Brazil and Paraguay [6] have developed regulatory approaches to forest conservation in agricultural landscapes. The effectiveness of these private-land forest regulations, and their potential as components of successful land strategies in other tropical nations, are poorly understood. Knowledge of their effectiveness is particularly important in light of the large number of tropical nations that are developing programmes for reducing their carbon emissions from deforestation and forest degradation [7]. In addition, there are uncertainties regarding the role of the Brazilian Forest Code (BFC) as a possible causal explanation of the steep decline in deforestation in the Brazilian Amazon since 2005 [4,7–9].

The BFC was designed to achieve forest protection on private rural landholdings. It establishes minimum percentages of private landholdings that must be kept under forest cover and also defines categories of ‘permanent preservation’ where economic activities are prohibited, including riparian zones, steep slopes and wetlands. The first version of the BFC was decreed as law in 1934 [10], establishing, for the first time, the responsibility of landholders to conserve forests, substituting the earlier view of forests as the object of utilitarian rights accruing to property holders. The 1934 BFC required that property owners maintain ‘protection forests’ serving a similar function as the permanent protection areas (in Portuguese, Areas de Proteção Permanente; APPs) of the modern BFC: to conserve hydrological functions, prevent soil erosion, support frontier defence, guarantee public health, protect sites of natural beauty and provide protection for rare native species of flora and fauna. Cutting of trees was strictly prohibited in these forests [11].

In 1965, the BFC was altered [12] to incorporate the concept of forests as a common good, imposing limits on the rights of property owners to alter their forests that were to be enforced through fines and other penalties. The 1965 BFC determined that the ‘legal reserve’ (LR) should constitute 50 per cent of the area of the property for properties located in the Legal Amazon and 20 per cent in other regions of the country. In 1989, the BFC was further amended to accommodate the agricultural expansion into the centre-west region of Brazil, particularly Mato Grosso (MT) [13]. We refer to this version of the BFC as ‘BFC 1989’.

The BFC has periodically become the focus of international attention because of the chronic tension between farm and livestock sectors that must retain forest on or restore forest to significant portions of their private landholdings, on the one hand, and environmentalists that view the BFC as the major policy instrument for preventing forest clearing outside protected areas, on the other hand. In 1996, in response to record high deforestation in the Amazon in 1995, the government adopted the temporary measure MP 1.511, increasing the LR on rural properties in the Amazon forest biome from 50 to 80 per cent, and prohibiting new clearing on properties already possessing ‘abandoned or underused’ areas or areas ‘used inappropriately with respect to the capacity of the soil’ [14]. We refer to this version of the law as BFC 1996. The MP 1.511 provoked an intense reaction from the agro-industrial lobby. Over the next 5 years, before the measure became law in 2001, it was modified repeatedly as the agricultural and environmental lobbies battled back and forth, leaving landholders in a state of chronic uncertainty regarding their legal obligations. Beginning in 2004 [15], heightened levels of enforcement of the BFC, especially in the Amazon region, contributed to a far more aggressive effort by the farm lobby to weaken the requirements that it imposes on landholders. During a bitter 2-year process beginning in 2010 that engaged the agro-industrial lobby, the Brazilian scientific community, the Brazilian socio-environmental community, farm organizations, international conservation groups and international diplomacy, the law was changed by the Brazilian Congress in 2012 [16,17]. We refer to this version of the law as BFC 2012.

The most significant changes found in BFC 2012 are the establishment of a minimum property size below which property holders are not obligated to establish a LR and the removal of the obligation to pay fines that were levied on landholders who had cleared forest prior to 2008. This amnesty from prior penalties is conditioned upon the registration of the property in state environmental cadastral systems and eventual compliance with the law, but provides greater flexibility to the landholder [18]. BFC 2012 also expands upon one of the mechanisms of the former law [19] designed to facilitate compliance by those landholders who have already cleared more forest than is legally permitted, allowing them to compensate for their excessive clearing through acquisition of the deforestation rights (i.e. essentially, the development rights) of another property in the same biome (formerly, this compliance mechanism could only be implemented within the same sub-basin).

Discussions of the BFC have been noteworthy for the paucity of information about the effectiveness of the policy on the ground. It is often assumed that a stricter LR requirement will lower deforestation rates, as expressed in the government's decision to increase the LR requirement from 50 to 80 per cent in 1996, following the record high deforestation in 1995. This change would only lower deforestation rates if (i) there were a shortage of forests that could be legally cleared (under the new 80% LR requirement) and (ii) landholders were summarily compelled to comply with the law. Compliance with the BFC, the effects of policy changes on this compliance, the cost implications of compliance for landholders and the environmental performance of agricultural landscapes that comply with the law are important issues that must be addressed to evaluate the BFC's effectiveness as an instrument of frontier governance.

In this study, we analyse farm- and watershed-level compliance with the BFC in the state of MT, Brazil's biggest agricultural producer, during the transitions from BFC 1989 to BFC 1996 and from BFC 1996 to BFC 2012. We then quantify the costs to landholders of compliance with the successive versions of the law in terms of opportunity costs associated with foregone rents from soya bean and beef production, and the costs of forest restoration. We also examine the environmental effects of full legal compliance with BFC 1989 versus BFC 1996 in terms of river discharge, forest carbon stocks and forest fragmentation within the Xingu River headwaters region, in northeastern MT.

## 2. Material and methods

### (a) Study area

Analyses of compliance with the BFC LR and associated economic costs to landholders of foregone rents from soya bean or beef over time were carried out for areas outside state and federal conservation areas, indigenous territories and other protected areas (including private-land APPs required under the BFC prior to 2012) within the Amazon forest biome of the state of MT. The state is located on the Amazon's agricultural frontier with more than half of the state (484 000 km^2^) lying within the Amazon forest biome. From 1996 to 2005, MT cleared an average of 7800 km^2^ of forest a year and accounted for just under 40 per cent of all deforestation in the Brazilian Amazon over that time period [20,21]. After 2005, annual deforestation in the state began to decline fairly steadily to reach a low of 777 km^2^ in 2012 [7,8,20].

To evaluate the environmental consequences of the change in BFC regulations, we focused on the 180 000 km^2^ Xingu River headwaters region in the northeastern part of the state where a rapidly expanding soya bean frontier, cattle ranching, indigenous lands and agrarian reform farm settlements are superimposed [22,23].

### (b) Assessing compliance

We assessed the degree of compliance with the BFC LR requirement in the forest biome of MT state. Analyses were conducted for 9459 properties in the MT environmental licensing system [24,25]. These properties had all been registered within the state environmental monitoring system by the end of 2011. This set of properties represents one third of MT's forest land area outside protected areas. Compliance was assessed at four critical dates: 1997 (1 year after the LR was elevated to 80%), 2001 (when the 80% LR was incorporated into law), 2005 (following a large surge in deforestation rates and marking the beginning of a period of heightened law enforcement) and 2009 (following 4 years of steep declines in deforestation and high levels of law enforcement). To better assess the spatial distribution of compliance and non-compliance across the state's entire forest biome, we used the boundaries of 9302 high-order sub-basins to serve as contiguous, wall-to-wall proxies for properties throughout the state. We focused only on the LR aspect of the BFC, because it represents a far larger area of forest than APP forests and includes the prime land for cultivation and livestock grazing. LRs thus represent a potentially more important source of income to individual landholders and the agricultural sector as a whole than APPs. Furthermore, the change in the LR percentage and the requirement to restore the LR were the primary concerns of rural landholders in the debate over changes in the legislation, especially in the Amazon region [26].

At each sample date (1997, 2001, 2005, 2009), we measured the forest area of each property and sub-basin using Prodes forest cover data [20]. We then determined the area to be restored to achieve compliance and/or the area of forest remaining in excess of the required amount for each property and basin. Official MT state deforestation data produced by the Secretary of Environment (SEMA-MT) were not available for all of the analysis dates, but a comparison of results of our analysis using Prodes and SEMA-MT data showed similar trends among the compliance categories (described in §3; electronic supplementary material, table S1). Although the Prodes system's minimum mapping unit is 6 ha, there is no evidence that this resolution influenced the results presented here. This area is larger than 1 per cent of properties in our analysis and may be larger than some annual deforestation events. However, we minimized the risk of missing cumulative deforestation within any given property over by examining 4-year periods. Furthermore, as the objective of the analysis is to estimate compliance with a national policy, use of official national forest cover data is appropriate.

To assess the extent to which the change in the BFC slowed illegal deforestation outside public (and other protected) lands in the forest biome, we estimated the amount and type (legal or illegal) of deforestation (outside public protected areas) in MT for three periods: 1997–2001, 2001–2005 and 2005–2009. For each period, properties and sub-basins were assigned to one of three categories in accordance with the LR requirements (50 or 80% of the property), as follows.

— *Legal deforestation***.** Forest clearing in properties and sub-basins that maintained at least 50 and 80 per cent forest cover, respectively, at both the beginning and the end of the period.
— *Illegal deforestation, new non-compliance*. Forest clearing in properties and sub-basins that had at least 50 or 80 per cent forest cover, respectively, at the beginning of the period but had less than these amounts by the end of the period.
— *Illegal deforestation, continued non-compliance*. Forest clearing in properties and sub-basins that had less than 50 or 80 per cent forest cover, respectively, at both the beginning and the end of the period.

### (c) Economic costs to landholders

To evaluate the economic implications for landholders of compliance with both the BFC 1989 (50% LR) and BFC 1996 (80% LR) requirements for each of the four dates, we estimated the opportunity cost of foregone rents resulting from the change in regulations. First, we assessed foregone rents associated with the reduction in lands available for conversion to agriculture caused by legal compliance. Second, we estimated the costs of restoring forest to come into compliance with the regulations, combining (i) the cost of restoring forests and (ii) the opportunity cost of the lands no longer available for agricultural production.

The opportunity costs of compliance with the BFC for each property and basin were estimated using spatially explicit rent models for soya bean production [15,27], beef cattle ranching [28] and sustainable timber harvest [29]. These are the three major economic activities in the region. These models estimate the potential rent of each economic activity through analyses of the costs of production (several of which are spatially dependent, such as transportation costs), yields and prices. For each of the three economic activities, the net present value (NPV) was estimated for 30 years into the future assuming a 5 per cent annual discount rate and a plausible schedule of highway paving (which affects transportation costs [30]). Agricultural land values are typically appraised by determining the production value of the land as determined by the NPV of the specific use to which that land is or will be put [31]. In this case, the range and distribution of NPV in the region is similar to that of actual land values for which prices are available at the municipal level [32]. Although the models do not account for short-term fluctuations, they use a set of assumptions that provide conservative projections of rent for each activity. The layers derived from the rent models were combined such that, for any given pixel, the NPV of timber harvests greater than zero (rotational logging in natural forests is permitted within LRs) was subtracted from the highest NPV value from either cattle or soya bean activities. Negative values resulting from this calculation were set equal to an NPV of zero. The resulting combined NPV map indicates the potential value of any given pixel, even if the pixel's current use is not known and/or the pixel is not being used in its rent-maximizing activity at the time.

At each date, we also estimated the costs of restoring LR areas to comply with the LR requirement under the BFC 1989, BFC 1996 and BFC 2012 versions of the BFC. We adapted the costs identified in field trials by organizations active in riparian zone restoration to estimate LR restoration costs, omitting methods that involve outplanting and maintenance of nursery-grown seedlings as this would be extremely costly and labour-intensive over the relatively large areas needing restoration. Restoration costs range from US$536 to 1327 ha^−1^ depending on the type of adjacent land use and the intensity of treatments required to restore forest (see the electronic supplementary material, table S2 [22]). Although the cost could be as low as US$0 ha^−1^ through natural regeneration, this can be slow and success would be highly dependent on the type and intensity of previous land use, as well as distance to nearest seed source.

### (d) Assessing the new Brazilian Forest Code

We calculated the cost implications for landholders of the most recent changes to the BFC (federal law 12.727, October 2012, BFC 2012). The amount of allowable clearing remains the same under the BFC 2012 (i.e. 20% on properties in the forest biome), but requirements for restoration of the LR are more flexible. The new regulations exempt properties from having to restore their LRs to 80 per cent if landholders have (i) complied with the requirements of previous iterations of the BFC (e.g. a property in the forest biome which maintained at least 50 per cent from before BFC 1996 to the present), and/or (ii) are less than 4 ‘fiscal units’ in size (i.e. between 120 and 400 ha in size, depending on their location within MT state). According to BFC 2012, these properties are not required to restore their LRs, but they may also not clear more forest. Under certain conditions, the requirement to restore the LR could be reduced to 50 per cent, if deemed appropriate by the state government. (iii) Properties located in counties (municípios) with half or more of their area occupied by protected areas (including indigenous lands), called ‘green counties’, may be required to restore only up to 50 per cent forest cover of the property. (iv) Additionally, states may require that properties located within specified zones (as determined by the approved state socio-economic and ecological zoning plan) restore only up to 50 per cent of their original forest cover. In neither (iii) nor (iv) may properties out of compliance with the 80 per cent LR clear new forest. We calculated the savings in area to be restored (and accompanying costs) under two alternative applications of the new BFC.

— Under *Scenario Alt1*, only properties meeting either of the first two criteria (i.e. less than 4 fiscal units in size and/or compliant under previous iterations of the BFC) were spared the requirement to reforest.
— Under *Scenario Alt2*, we added properties located either in green counties or in designated socio-economic zones, reducing the restoration requirement to 50 per cent.

For each scenario, we assessed the area to be restored, opportunity costs and the costs of restoration as described above. We also assessed the potential for compensating the area to be restored by designating a comparable area remaining to be legally cleared in the state as a means of reducing the cost of restoration. We calculated the reduction in on-farm restoration (and associated costs) that could be achieved by purchasing the deforestation rights from those lands having forest cover over and above the required 80 per cent.

### (e) Ecological consequences

To assess the ecological consequences of each of the three versions of the BFC, assuming perfect compliance with each, we adapted a previously developed dynamic landscape model [22,23] for the Xingu headwaters region that simulates land cover under the alternative scenarios. The assumptions of the alternative scenarios are as follows.

— *BFC 1989*: deforestation up to 50 per cent of the property, outside riparian buffer areas, is permitted; if forest cover is below 50 per cent, restoration up to 50 per cent is required; LR is calculated in addition to riparian zone buffer areas (50 m on either side of streams owing to 100 × 100 m spatial resolution of model).
— *BFC 1996*: deforestation up to 20 per cent of the property, outside riparian buffer areas, is permitted; if forest cover is below 50 per cent, restoration up to 80 per cent is required; LR is calculated in addition to riparian zone buffer areas.
— *BFC 2012*: deforestation up to 20 per cent of the property, outside riparian buffer areas but including existing riparian forest in the total forest area of the property, is permitted; if forest cover is below 80 per cent, restoration up to 80 per cent is required, except if the conditions described for the BFC 2012 *Alt1* and *Alt2* scenarios are met: in these cases, either no further restoration is required (*Alt1*) or restoration up to 50 per cent is required (*Alt2*).

For each of the three BFC scenarios simulated for the Xingu headwaters, ecological consequences were assessed for the entire region, including all protected areas. We compared the landscapes in terms of carbon stocks, river runoff from the Xingu main stem at the MT–Pará border and habitat fragmentation [22,23]. Indicators were assessed as described in [23].

## 3. Results

### (a) Compliance

In 1997, shortly after the LR was increased from 50 to 80 per cent in the Amazon forest biome (BFC 1996), 21 per cent of properties in the forest biome had less than 50 per cent forest cover and were, therefore, not in compliance with the BFC 1989 (see the electronic supplementary material, table S3). Forty-two per cent of the properties had less than 80 per cent forest cover in 1997 and were not in compliance with the new law (BFC 1996). By 2001, this percentage had grown to 67 (table 1). Of these, 86 per cent were properties that continued to be non-compliant with the 80 per cent LR requirement, whereas the remainder shifted from compliant to non-compliant (table 1). In 2001, nearly half of properties remained in compliance with the previous 50 per cent LR requirement. Just over three-quarters (78%) of properties that cleared illegally in the time period relative to the 80 per cent requirement were also violating the 50 per cent requirement.

**Table 1.** Classification of registered properties and sub-basins by percentage forest cover and compliance with Brazilian Forest Code (BFC). Evolution of compliance with two successive iterations of the BFC's legal reserve (LR) requirement (for the Amazon forest biome) for three time periods: (i) BFC 1989 (50% LR): property must maintain or restore up to at least 50% forest cover; (ii) BFC 1996 (80% LR): property must maintain or restore up to at least 80% forest cover. Illegal deforestation and legal deforestation refer to those properties and sub-basins that were above or below, respectively, the maximum percentage of LR clearing allowed by the BFC. Continued non-compliance refers to properties and sub-basins that began the time period out of compliance. New non-compliance refers to properties and sub-basins that moved from compliance to non-compliance during the time period. All analyses are presented for a set of properties registered in the state environmental licensing programme, as well as for the entire state's forest biome area outside protected areas.

	period 1 (1997–2001)				period 2 (2001–2005)				period 3 (2005–2009)			
	BFC 1989 (50% LR)		BFC 1996 (80% LR)		BFC 1989 (50% LR)		BFC 1996 (80% LR)		BFC 1989 (50% LR)		BFC 1996 (80% LR)	
	area (km^2^)	*n*	area (km^2^)	*n*	area (km^2^)	*n*	area (km^2^)	*n*	area (km^2^)	*n*	area (km^2^)	*n*
*registered properties*												
illegal deforestation												
continued non-compliance	1058 (16%)	4596 (42%)	3320 (50%)	5462 (58%)	1291 (12%)	4863 (51%)	4351 (40%)	6311 (67%)	497 (23%)	6254 (66%)	1302 (62%)	7737 (82%)
new non-compliance	2970 (45%)	869 (9%)	2742 (41%)	849 (9%)	5231 (48%)	1391 (15%)	5769 (53%)	1426 (15%)	786 (37%)	329 (3%)	645 (30%)	218 (2%)
legal deforestation												
in compliance	2612 (39%)	4596 (49%)	576 (9%)	3148 (33%)	4411 (40%)	3205 (34%)	813 (7%)	1722 (18%)	834 (39%)	2876 (30%)	170 (8%)	1504 (16%)
*whole forest biome (outside protected areas)*												
illegal deforestation												
continued non-compliance	4364 (21%)		12 199 (57%)		5473 (18%)		16 601 (55%)		2619 (35%)		5812 (79%)	
new non-compliance	6234 (29%)		6583 (31%)		10 366 (34%)		10 461 (35%)		1427 (19%)		869 (12%)	
legal deforestation												
in compliance	10 631 (50%)		2447 (12%)		14 211 (47%)		2987 (10%)		3354 (45%)		719 (10%)	

By 2005, following a spike in deforestation from 2002 to 2004, 82 per cent of properties were not in compliance with the BFC 1996. From 2001 to 2005, 68 per cent of properties remained non-compliant and 15 per cent entered non-compliance with the 80 per cent LR requirement. By 2005, over 80 per cent of those properties with illegal clearing in the time period under the 80 per cent requirement were properties that were also illegal by the 50 per cent requirement. Finally, in the period from 2005 to 2009, when deforestation plummeted [7,8], only an additional 3 per cent of the properties became non-compliant (table 1). Almost all new illegal clearing was caused by properties not considered to be compliant even under the 50 per cent LR requirement.

A similar analysis using sixth order and higher sub-basins instead of properties provides a picture of aggregate and spatial trends in compliance (table 1). Overall, the sub-basin-level analysis exhibited trends consistent with those for the property-level analysis. However, as a rule, the proportion of legal deforestation (by area and number of analysis units) was higher in the sub-basin analysis (table 1). This is likely a result of the under-representation of areas that remained forested throughout the analysis within the set of registered properties, because the registered properties are clustered in areas closer to the frontier and not randomly distributed throughout MT's forest biome (see the electronic supplementary material, figure S1).

**Figure 1.**
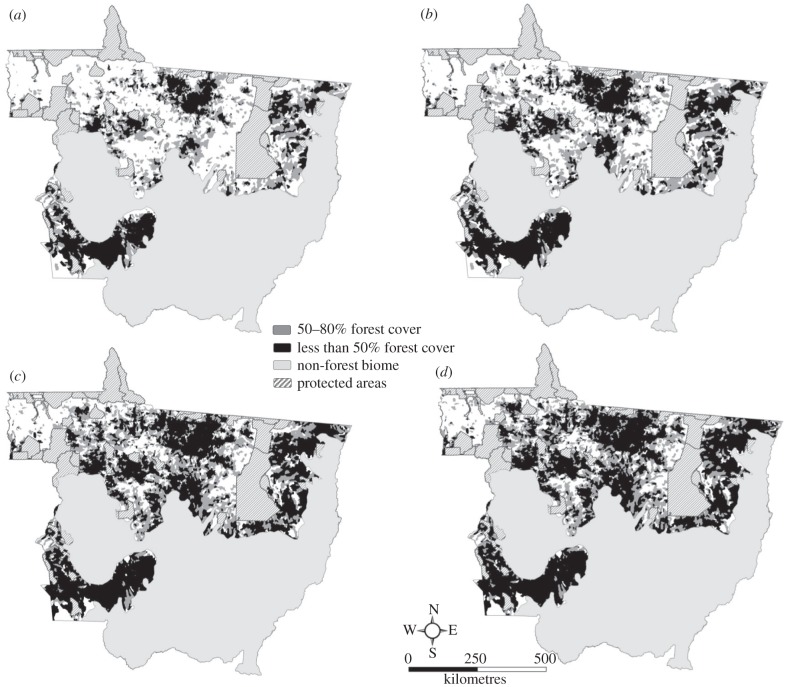
Distribution of sub-basins (which serve as proxies for private properties) in the forest biome of Mato Grosso state that were out of compliance with two iterations (50 and 80% LR requirement) of the Brazilian Forest Code at four time points: (*a*) 1997, (*b*) 2001, (*c*) 2005 and (*d*) 2009. Sub-basins with less than 50% forest cover (black), 50–80% forest cover (grey) are highlighted. Areas with no shading indicate areas under private ownership with more than 80% forest cover.

During the period from 1997 to 2001, a total of 21 229 km^2^ of forest were cleared (table 1). Under the 80 per cent LR requirement, only 12 per cent was cleared legally. Fifty-seven per cent of total clearing in the period took place in sub-basins already non-compliant in 1997, and 31 per cent in sub-basins that became newly non-compliant over the 4 years (table 1). The increase in non-compliant sub-basins was largely on the eastern side of the Xingu River basin and in the northern section of the BR-163 (Cuiabá–Santarém) highway (figure 1*a,b*), an area where soya bean production was undergoing a boom during this period [8,21].

The area of forest cleared rose by 41 per cent from 2001 to 2005, reaching a total of 30 049 km^2^. Ninety per cent of this clearing was illegal; approximately 40 per cent of this illegal clearing occurred in sub-basins that became non-compliant for the first time during this period. Deforestation declined dramatically from 2005 to 2009, totalling only 25 per cent (7400 km^2^) of the area cleared in the previous 4 years. Compared with the previous two periods, a similar proportion of clearing was illegal. However, only 12 per cent of illegal clearing took place in sub-basins that were still compliant in 2005. Over this period, the major increase in non-compliance occurred on the western side of the Xingu River basin and to the west of the BR-163 highway, also largely coinciding with the area of soya bean expansion, which was beginning to displace timber extraction. By 2009, the only major regions of forest outside protected areas that were still in compliance with the 80 per cent LR requirement were along the western side of the Xingu Indigenous Park and in the far northwest of the state, near the borders with Rondônia and Amazonas states (figure 1*d*).

## 4. Costs to landholders of compliance

### (a) Opportunity cost of foregone rights to clear forest

The increase in the legal forest reserve requirement from 50 to 80 per cent imposed substantial costs on landholders. The first cost that we consider is that associated with foregone rents from soya bean or cattle ranching incurred through compliance with the mandatory forest LR requirements; that is, with the maintenance of those forests that were legal to clear under BFC 1989 and became illegal to clear under BFC 1996 (table 2). This is the area of forest, in aggregate, in excess of 50 per cent of each property minus the forest area in excess of 80 per cent of each property (forest in excess of 80% could still be legally cleared under BFC 1996). In 1997, when the law changed, the opportunity cost of foregone rights to clear forests in the registered properties having forest cover in excess of the 50 per cent requirement amounted to approximately US$1.2 billion or an average of US$227 000 per landholder. In the aggregate for the entire forest biome, we estimate that the change in the regulation represented an opportunity cost of foregone rights to clear forest of US$3 billion in potential rents from soya bean farming or cattle ranching (table 2). These opportunity costs declined over time as landholders illegally cleared their private forestlands.

**Table 2.** Potential value of forest lands remaining to be cleared legally in Mato Grosso state under two alternative legal reserve (LR) size requirements of the Brazilian Forest Code (BFC) at four key dates: (i) BFC 1989 (50% LR): property must maintain or restore up to at least 50% forest cover; (ii) BFC 1996 (80% LR): property must maintain or restore up to at least 80 per cent forest cover. The opportunity cost of the change in LR size at each date is also presented. All analyses are presented for a set of properties registered in the state environmental licensing programme, as well as for the entire state's forest biome area outside protected areas.

	1997		2001		2005		2009	
	BFC 1989 (50% LR)	BFC 1996 (80% LR)	BFC 1989 (50% LR)	BFC 1996 (80% LR)	BFC 1989 (50% LR)	BFC 1996 (80% LR)	BFC 1989 (50% LR)	BFC 1996 (80% LR)
registered properties (*n* = *9459*)								
number of properties with forest cover in excess of requirement	5096 (54%)	3673 (39%)	4233 (45%)	2823 (30%)	2832 (30%)	1412 (15%)	2504 (26%)	1192 (13%)
area remaining to be cleared (km^2^)	26 903	7716	22 565	6195	14 963	3392	13 693	3024
potential value of lands remaining to be cleared (million US$)	1611	454	1296	349	773	169	707	151
opportunity cost of change in regulation (million US$)	1157		947		604		557	
average opportunity cost per affected landholder (US$)	226 900		223 717		213 194		222 274	
whole forest biome (outside protected areas)								
area remaining to be cleared (km^2^)	78 573	21 998	64 420	16 881	44 317	9740	40 241	8646
potential value of lands remaining to be cleared (million US$)	4079	1067	3237	775	2056	411	1879	370
opportunity cost of change in regulation (million US$)	3012		2462		1645		1509	

### (b) Costs of re-establishing mandatory forest cover

In addition to not clearing beyond the permitted percentage of an individual property, landholders were required to restore the LR to meet the regulations. The cost of such restoration is represented by both the additional foregone rents from the land that can no longer be used for activities such as soya bean and cattle ranching, as well as by the direct costs of forest restoration. With the change in regulations, increasing the LR from 50 to 80 per cent in 1996, the total area to be restored by the 9459 properties increased by over 10 000 km^2^ (table 3). The share of properties required to restore forest cover increased from 42 to 58 per cent, whereas the total cost of restoration increased by nearly US$1.7 billion. Most of this total (58%) is due to the costs of restoration or regeneration, rather than to the opportunity cost of taking those lands out of production. The cost of restoration could be lower if natural regeneration is allowed to proceed unaided (table 3). By 2009, the total area to be restored to forest increased to nearly 31 000 km^2^. The share of properties with some deforested area to restore had increased to 84 per cent of all properties analysed (table 3), at a potential cost of over US$5.1 billion. For the whole forest area of MT (using the sub-basin analysis), we estimate that the change in regulation in 1996 initially led to an increase of over 32 000 km^2^ in the total area requiring restoration and an increase in cost of US$5.2 billion. By 2009, the total area requiring restoration under the 80 per cent LR had nearly doubled to 95 000 km^2^, with a cost of approximately US$15 billion (table 3).

**Table 3.** Cost of forest restoration for lands to be restored to full forest cover in order to come into compliance with the Brazilian Forest Code (BFC) legal reserve (LR) regulations under alternative sets of requirements: (i) BFC 1989 (50% LR): property must maintain or restore up to at least 50 per cent forest cover; (ii) BFC 1989 (80% LR): property must maintain or restore up to at least 80 per cent forest cover; (iii) BFC 2012 (*Alt1)*: property must maintain or restore up to at least 80 per cent of forest cover, unless property is smaller than 4 ‘fiscal units’ (120–400 ha) in size or complied with previous versions of BFC; and (iv) BFC 2012 (*Alt2)*: property must maintain or restore up to at least 80 per cent of forest cover, unless it meets the conditions of *Alt1* and/or the state stipulates that it may restore up to only 50% because it falls in an eligible zone. All analyses are presented for a set of properties registered in the state environmental licensing programme, as well as for the entire state's forest biome area outside protected areas.

	1997		2001		2005		2009		BFC 2012	
	BFC 1989 (50% LR)	BFC 1996 (80% LR)	BFC 1989 (50% LR)	BFC 1996 (80% LR)	BFC 1989 (50% LR)	BFC 1996 (80% LR)	BFC 1989 (50% LR)	BFC 1996 (80% LR)	*Alt1*	*Alt2*
properties (*n* = *9459*)										
number of non-compliant properties	3994 (42%)	5462 (58%)	4863 (51%)	6311 (67%)	6254 (66%)	7737 (82%)	6583 (70%)	7955 (84%)	2993 (32%)	2686 (28%)
area to be restored (km^2^)	5257	15 711	7557	20 828	10 889	28 960	11 736	30 708	14 947	13 134
opportunity cost—restoration (million US$)	375	1098	563	1501	841	2138	894	2240	1083	950
cost of restoration (million US$)	490 (±208)	1464 (±621)	704 (±299)	1940 (±824)	1014 (±431)	2698 (±1145)	1093 (±464)	2861 (±1215)	1392 (±591)	1223 (±520)
total cost—restoration (million US$)	865	2562	1267	3441	1855	4836	1987	5101	2475	2173
whole forest biome (outside protected areas)										
area to be restored (km^2^)	17 413	50 110	24 488	66 222	34 435	89 130	37 758	95 436	83 100	76 867
opportunity cost—restoration (million US$)	1171	3393	1702	4486	2402	6014	2584	6332	5642	5173
cost of restoration (million US$)	1622 (±689)	4668 (±1982)	2281 (±969	6169 (±2619)	3208 (±1362)	8303 (±3525)	3517 (±1493)	8890 (±3774)	7741 (±3287)	7160 (±3040)
total cost—restoration (million US$)	2793	8061	3983	10 655	5610	14 137	6101	15 222	13 383	12 333

### (c) Implications of the new Brazilian Forest Code (BFC 2012)

In October 2012, the Brazilian government approved a new BFC that reduces or eliminates the requirement to restore the LR on some properties [17]. Using 2009 forest cover as a basis for comparison, we estimate that the new BFC could reduce the restoration burden throughout MT's forest biome by 12 000–18 000 km^2^, depending on the extent to which restoration requirements are relaxed under the new regulations (table 3). The cost savings over the previous regulations would be about US$2.5–3 billion.

We also considered the reduction in restoration costs that could be obtained by purchasing the deforestation rights of other properties in the state with more than the required amount of vegetation on their land [6]. For the 2009 landscape, the area remaining to be legally cleared and, therefore, available for such a trade was 8646 km^2^ (table 4). Under BFC 2012 *Alt1*, this trade would reduce the restoration burden to approximately 75 000 km^2^, and under BFC 2012 *Alt2* to 68 000 km^2^. This would reduce the direct costs of restoration (i.e. outplanting) by approximately US$8 million. The opportunity cost could be reduced by approximately US$585 million. As the lands remaining to be legally cleared have a potential NPV for soya bean or beef production of approximately US$370 million, purchasing the deforestation rights in lieu of reforesting on-farm could lead to an overall savings of US$1 billion or more.

**Table 4.** Estimated costs of reducing deforestation through trade of deforestation rights within the forest biome of the state of Mato Grosso under the new Brazilian Forest Code regulations. Area of lands to be restored and available for trade is based on 2009 land cover.

	BFC 2012 scenarios	
	*Alt1*	*Alt2*
area to be restored (km^2^)	83 100	76 867
average value of lands to be restored (US$ ha^−1^)	679	673
average cost of restoration (US$ ha^−1^)	932	932
area remaining to be legally cleared (km^2^)	8646	8646
value of lands remaining to be cleared legally (million US$)	370	370
area to be restored after trading within state (km^2^)	74 454	68 221
savings in restoration cost (million US$)	806	806
savings in opportunity cost of lands to be restored (million US$)	587	582
total savings from deforestation rights trade (million US$)	1023	1018

Under the new regulations, the geographical range for trading deforestation rights has been broadened to include the entire Amazon forest biome. Thus, the remaining area to be reforested under either scenario could theoretically be traded for lower value lands elsewhere in the Amazon, such as Amazonas state, where land prices are far lower than in Mato Grosso [15].

### (d) Ecological consequences

**Figure 2.**
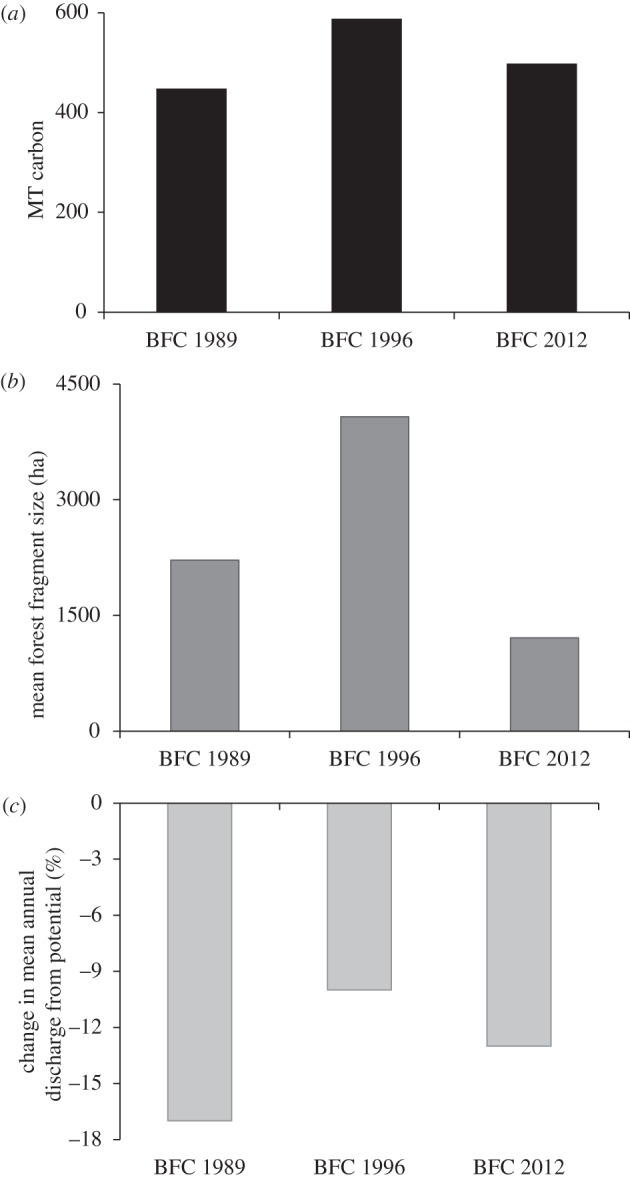
Indicators of potential environmental performance for three successive iterations of the Brazilian Forest Code (BFC 1989, BFC 1996 and BFC 2012) for the Xingu River headwaters region of northeastern Mato Grosso: (*a*) total carbon stocks stored in forest vegetation; (*b*) mean forest fragment size; (*c*) change in mean annual discharge from control scenario.

Our simulations of the potential long-term effects of the three versions of the BFC found that BFC 2012, if fully implemented, would provide ecological benefits that are intermediate to the other versions (see figure 2 and electronic supplementary material, table S4). BFC 1989, with the 50 per cent LR requirement, results in the lowest forest carbon stocks, the highest stream discharge, but intermediate forest fragment size. BFC 1996, with 80 per cent LR requirement, would achieve the highest carbon stocks, the lowest discharge and the largest fragments, if implemented. The larger fragment size in both the BFC 1989 and the BFC 1996 scenarios is due to restoration requirements.

## 5. Discussion

The BFC was designed to protect public interests in private-land forests, an intent that was fully canonized in the new constitution of 1988. To successfully protect the ‘social function’ [33] of forests on private lands, the code must change the behaviour of landholders. They must comply with the restrictions on forest clearing that are defined by the code, and adjust this behaviour when the code changes. The main conclusion of this study is that compliance with the BFC of 1989 was moderate (50%) shortly after the decree establishing the new LR at 80 per cent and declined to 10 per cent compliance with the new BFC (1996) by 2009. The farm sector of MT's Amazon forest region became predominantly illegal in the course of 12 years.

We found no evidence that changes to the BFC to make it more restrictive (80% versus 50% LR) inhibited deforestation. In a scenario of full legal compliance, deforestation would have halted on properties that had already reached 20 per cent forest clearing when the law was changed in 1996, continuing only on those properties with more than 80 per cent forest cover remaining. In this full compliance scenario, only 22 000 km^2^ of forest in MT were available for legal clearing when the LR was raised to 80 per cent. In practice, however, the level of compliance with the 80 per cent legal forest reserve requirement declined to 12 per cent in 2001 and stabilized at 10 per cent from 2005 (following the surge in clearing through 2004) to 2009. Despite the more restrictive BFC, deforestation in the state climbed from a total of just 20 000 km^2^ for the 1997–2001 period to approximately 30 000 km^2^ for the 2001–2005 period, a further indication that the code revision had little if any inhibitory influence on forest clearing by landholders.

It is perhaps not surprising that the level of compliance with the new LR requirement after 1996 was low. Compliance with environmental regulation is highest when (i) the process by which landholders can achieve compliance is clear and practical, (ii) the probability of non-compliant landholders being identified is high, (iii) the probability of apprehended landholders being punished (i.e. by paying fines or facing imprisonment) is high, (iv) the costs of compliance are low and (v) there are positive incentives for compliance [34,35]. In sum, compliance is highest when non-compliance is very expensive and/or when compliance brings tangible benefits. This study contributes to a growing literature documenting that these conditions were not adequately met by the BFC [13,25,36–39].

The change in the BFC was not accompanied by an effective programme for helping landholders bring their properties into compliance if they had already exceeded 20 per cent clearing. It was only in 2001, when the 80 per cent LR requirement became law, that mechanisms for facilitating compliance were created. Properties could be brought into compliance through restoration of forests to bring the forest cover up to 80 per cent of the property, compensation of the LR through the purchase of forest development rights on other properties [13,19] and payment into a fund that would maintain or expand state protected areas [13]. These mechanisms were not adequate nor were they implemented or enforced. Even in 2005, only 13 000 km^2^ of forest could have been cleared legally or could have been set aside to compensate non-compliant properties. However, the area of illegal clearing by this year had reached 74 000 km^2^, more than five times the area available for compensation. Very few landholders brought their properties into compliance through compensation on other properties; between 1999 and 2007, only five such applications were processed by MT's environmental agency [13]. The state attempted to establish a fund that landholders could pay into as compensation for excessive forest clearing, but the national Ministry of Environment rejected this fund [13]. Finally, the state's ecological-economic land-use zoning plan, which has been under development for over 20 years, could have facilitated compliance by relaxing the restoration requirement in some zones; however, it is under judicial dispute and has yet to be implemented [13].

Compliance with the BFC 1996 may also have been low because of landholder uncertainty about whether it would be maintained. We identify two major sources of this uncertainty. First, MT declared in 2000 that the LR requirement for the ‘transition forests’, which include over half of the forests in the state, was only 50 per cent, despite the change to 80 per cent for all forests in the Legal Amazon in the federal BFC. This state-level declaration went unchallenged by the federal government for 5 years, and was accompanied by a vigorous debate about the definition of transition forest. In 2005, the federal government over-ruled the state interpretation, and the entire forest biome returned to an 80 per cent LR requirement. During this period, many forest clearing permits had been issued to landholdings to maintain only a 50 per cent LR [13].

A second source of uncertainty regarding the longevity of the 80 per cent requirement was the frequent attacks on the BFC within the Brazilian National Congress. The bancada ruralista (ruralist constituency, primarily the agricultural lobby) advanced proposals to reduce the LR requirement of the BFC almost annually [18,36]. The reduction of the LR requirement (or at minimum, reduction of the requirement to restore vegetation up to the LR requirement) was an important plank in the political platform of politicians representing agro-industry states and/or regions, agro-industry's representative organizations, such as FAMATO (MT Agriculture Foundation) and CNA (National Agriculture Confederation), and may have given landholders a sense of impunity regarding the BFC [13]. Since 2005, MT's agro-industrial sector has continued to seek ways to reduce the LR and/or to legalize properties that cleared in excess of the permitted amount without requiring restoration [13]. In 2010, the proposal to provide amnesty to properties having cleared native vegetation (legally or illegally) through mid-2008 was successfully brought before Congress, and ultimately approved in 2012 [17].

In 2000, MT launched an environmental licensing system for private rural properties (Sistema de Licenciamento Ambiental para Propriedades Rurais; SLAPR) as a means of monitoring compliance with the BFC and of differentiating between legal and illegal deforestation [13,37]. Although INPE had been monitoring deforestation in the Amazon (PRODES) since 1988, the system was unable to discriminate legal and illegal clearing for want of cadastral data. Analyses of the system indicate that deforestation (including illegal deforestation) on properties registered in the system actually exceeded that of properties outside the system [13,25,36]. Furthermore, even when violators identified by the system were fined, only a small fraction (1% or less) of those fines were collected [39,40]. Often, the fines were cancelled or remained pending under legal challenges for several years for reasons ranging from unclear land title to graft to regulatory error [39,40].

These weaknesses in implementation of the BFC may have been reinforced by the costs incurred by landholders through compliance with the code. The costs of registering with SLAPR alone have been demonstrated to be prohibitive to many landholders, particularly if the landholder attempts to maintain a LR of 80 per cent [13,25,38]. As these studies suggest, opportunity costs present an even greater obstacle to compliance. The foregone rents from deforestation-dependent economic activities, such as cattle ranching and soya bean cultivation, were particularly strong incentives for landholders to clear more forest than allowed under the BFC, taking the risk of getting caught and paying fines because of the high potential rents. We estimate that the aggregate opportunity cost incurred by landholders through the increase in the LR requirement in 1997 was approximately US$3 billion.

Although compliance with the change in the LR requirement in 1997 was low, simulation modelling allows us to understand the potential ecological benefits of this and the subsequent (BFC 2012) policy decision if fully implemented. The higher level of vegetation cover associated with the 80 per cent LR scenario relative to the 50 per cent scenario signifies a lower potential for surface runoff and associated soil erosion [23], and lower potential for stream and river flooding [41]. It also implies higher evapotranspiration, which reduces the likelihood of deforestation-driven changes in the regional rainfall system, which some observations [42] and models suggest can take place when clearing exceeds 60–70 per cent of the original forest cover [43–45]. The southeastern Amazon is likely to be severely impacted by rainfall reduction through climate change [46], and the maintenance of high levels of evapotranspiration could diminish the likelihood of these changes in rainfall [47]. Improvements in water quality and habitat under a fully implemented BFC (including the post-2012 version) could have direct positive impacts on the livelihoods of the indigenous peoples who reside in the Parque Indigena do Xingu (Xingu Indigenous Park) located at the core of the Xingu headwaters region. Tribes such as the Kisedje have observed declines in the quality of the fish and turtles that they catch for subsistence consumption and have also noted changes in the timing and strength of rains at the beginning of the rainy season (Chief C. Kisedje 2005, personal communication).

Although not retaining as much forest as mandated under a fully implemented BFC 1996, the BFC 2012 also retains 50 million tonnes of carbon (180 million tonnes of CO_2_ equivalent (tCO_2_e)) more than the 50 per cent LR landscape of BFC 1989. Carbon storage is the only ecosystem service in the Amazon region that is close to having a robust compensation mechanism to provide incentives for its maintenance. With reduced emissions from deforestation and forest degradation policies and programmes under development nationally and at the state level (including in MT) [7], carbon could become a promising positive incentive for offsetting the costs of enforcement so that landholders comply with the new legislation. These incentive mechanisms are stalled within the UN Framework Convention on Climate Change, but are being developed by California state (USA), Australia and elsewhere [7].

In sum, MT state's experience provides insights into the limits of command-and-control regulations designed to defend public interests in private-land resources. The change in the LR requirement from 50 to 80 per cent of private properties imposed US$2–3 billion in forgone potential present and future rents on the region's farmers and ranchers, and was ineffective in creating processes and procedures through which landholders who wished to comply with the law could do so [13,25], nor was a programme developed to provide positive incentives for landholders to comply with the BFC (but note that the BFC 2012 now requires that state and federal governments develop a multi-faceted incentives programme to promote compliance). The validity of the 80 per cent LR requirement was undermined by the state's decision that the transition forests of the region had a 50 per cent LR requirement, by the frequent attacks on the BFC within the Brazilian legislature by the agricultural lobby and by the low levels of enforcement of the 80 per cent rule. Ironically, non-compliance was also reinforced by the perceived risks associated with compliance. Because of the threat that compliance poses to the culture of graft and corruption, law enforcement officers punish those making an effort to comply with the law [48].

The recent struggle over the BFC culminating in changes that facilitate farm compliance is best understood in the context of the evolution of the policy itself, governmental enforcement of the BFC and market conditions underway from 2005 to 2012. This was a period of dramatic increases in both governmental efforts (i) to enforce the BFC, as well as (ii) market rejection of landholders who were actively clearing their land, as manifested in the soya bean and beef moratoria and the new international standards developed under the Round Table on Responsible Soy (RTRS) and other roundtables [15,49]. MT's powerful soya bean growers' organization, Aprosoja, abandoned the RTRS in 2009 when a mechanism for compensating landholders for the high costs of legal compliance with the BFC 1996 had still not been developed [50]. Legal enforcement was supported by two new governance mechanisms. The SLAPR was supplemented by the Cadastro Ambiental Rural (CAR; rural environmental registry) within a state land legalization programme called ‘MT Legal’. This programme provided a legal grace period for non-compliant properties that deforested illegally up to July 2007. In 2008, the federal government initiated a ‘black list’ programme that suspended access to agricultural credit programmes for properties in counties that had the highest rates of deforestation and the lowest percentage of their private properties within the CAR [15].

## 6. Conclusion

This case study identifies a crucial challenge for lawmaking designed to defend public interests in privately controlled natural resources. At its core, it illustrates one legislative attempt to reconcile a trade-off that has been repeated throughout human history. Is maintaining ecological integrity, higher evapotranspiration, carbon stocks, greater rainfall security, reduced soil erosion and the maintenance of native habitats over the state of MT worth several billion dollars in lost rents? If the answer is affirmative, then creating incentives that facilitate private landholders' compliance with the law would seem to be important. The BFC is a piece of innovative legislation, and one of the first to recognize and attempt to protect the broader public interests in private land forests in the tropics. It has great potential for fostering the reconciliation of conservation with agricultural development, but in its current state, that potential is not being realized. The Brazilian government might have achieved the objectives of defending public interests in private forests if the shift to 80 per cent legal reserve had been implemented in a different way. First, the change should have been accompanied by an effective set of options through which landholders could bring their properties into compliance with the new law. Second, the government should have developed a system of positive incentives for complying with the new regulation, potentially including compensation of at least part of the opportunity cost associated with forgone rents from soya bean cultivation or cattle ranching. The carbon market represents an important opportunity to achieve these economic incentives and may be necessary to secure Brazil's historic progress in lowering deforestation to more than 75 per cent below its 10-year average, reducing global greenhouse gas emissions by 1.8 per cent [7]. The farm sector must do its part, investing in the design and implementation of the new law so that it includes a legal framework for developing effective positive incentives for privately held forests.
